# Assembly, Characterization and Comparative Analysis of the Complete Mitogenome of Small-Leaved *Eriobotrya seguinii* (Maleae, Rosaceae)

**DOI:** 10.3390/genes17010107

**Published:** 2026-01-20

**Authors:** Muhammad Idrees, Fardous Mohammad Safiul Azam, Meng Li, Zhiyong Zhang, Hui Wang, Yunyun Lv

**Affiliations:** 1 College of Life Science, Neijiang Normal University, Neijiang 641000, China; zhangzyong219@126.com (Z.Z.); whscnj@126.com (H.W.); lvyunyun_sci@foxmail.com (Y.L.); 2 Department of Biotechnology and Genetic Engineering, Faculty of Life Sciences, University of Development Alternative, Dhaka 1209, Bangladesh; 3 Co-Innovation Center for Sustainable Forestry in Southern China, College of Biology and the Environment, Nanjing Forestry University, Nanjing 210037, China; limeng@njfu.edu.cn

**Keywords:** assembly, mitochondrial genome, *Eriobotrya seguinii*, Rosaceae, China

## Abstract

Background. *Eriobotrya seguinii* (Lév.) Cardot ex Guillaumin (Rosaceae, Maleae) is native to China and inhabits various altitudes within the subtropical biome of the Yunnan-Guizhou Plateau. The complexity of the plant mitogenome has impeded a systematic description of this species, leading to a limited understanding of its evolutionary position. Methods. In this study, we constructed, annotated, characterized, and compared the complete *E. seguinii* mitogenome with previously reported *Eriobotrya japonica*. Results. The *E. seguinii* mitogenome exhibited a typical circular architecture, spanning 372,899 bp in length, with a GC content of 46%, making it the smallest and highest GC content of any known *Eriobotrya* species. It encodes 71 unique genes, comprising 47 protein-coding genes, 20 transfer RNA (tRNA) genes, and 4 ribosomal RNA (rRNA) genes. The genome contains rich repetitive sequences, with mononucleotides, A/T bias, and forward and palindromic repeats being the most prevalent. The predominant codons were GCU (Ala) and UAU (Tyr), with frequencies of 1.54 and 1.53, respectively. Thirteen genes (*atp9*, *atp6*, *atp1*, *rps14*, *sdh4*, *sdh3*, *rps12*, *rnaseH*, *nad1*, *nad6*, *nad7*, *rpl16*, and *mttB*) demonstrated high *Pi* values, ranging from 0.84 to 1. The evolutionary lineage of *E. seguinii* was explored using mitogenome data from 19 genera within the Rosaceae family, revealing that *Eriobotrya* species are monophyletic and closely related to *E. japonica* (MN481990). Conclusions. Understanding the mitogenome characteristics of *E. seguinii* enhances our understanding of its genesis and classification based on mitochondrial genome data. This study provides additional evidence for future research on the evolutionary relationships among species in the Rosaceae family.

## 1. Introduction

*Eriobotrya seguinii* (Lév.) Cardot ex Guillaumin (Small-leaved loquat, Chinese 小叶枇杷) is a small tree or shrub, characterized by oblong to oblanceolate leaves with incurved crenate margins, 10 pairs of lateral veins, abaxially villous when young, and glabrescent when mature. The panicle, peduncle, and pedicel are densely covered with rusty tomentose. The ovary is villous apically, with two ovules per locule; the styles are three or four (villous at the base), the stamens are fifteen, and the pome is somewhat pubescent with persistent sepals. This species is native to China and thrives in the subtropical biome at low to high elevations on the Yunnan-Guizhou Plateau [1].

*E. seguinii* was first described in the *Repertorium Specierum Novarum Regni Vegetabilis* in 1912 under the genus *Symplocos* Jacq. [2]. In 1918, Cardot transferred the name from *Symplocos* to *Eriobotrya*, renaming it *E. pseudoraphiolepis*, a superfluous nomenclature [3]. Guillaumin [4] proposed an alternative name that is currently recognized as valid [5]. In a recent taxonomy, the genus *Eriobotrya* was considered a synonym within the broadly circumscribed genus *Pyrus* [6] and subsequently retreated under *Rhaphiolepis* [7]. Consequently, the evolutionary relationships within the *Eriobotrya* genus remain a significant concern in terms of plant taxonomy. Regional revisions and floral treatments [1,8,9,10,11] as well as molecular and morphological evidence treating *Eriobotrya* as a separate, distinct genus have garnered broad support among botanists [12,13,14,15,16,17,18].

Few studies have been conducted on the mitogenomes of Rosaceae species, despite recent advancements in nuclear and chloroplast genomic sequencing [19,20,21,22,23]. Many *Rosaceae* species, including those in the genus *Eriobotrya*, are under-represented, emphasizing the need for additional sequencing of unreported species to better understand the evolutionary relationships. Currently, the National Center for Biotechnology Information database (NCBI: https://www.ncbi.nlm.nih.gov/, accessed on 5 August 2025) deposited 21 genera of Rosaceae mitogenomes, though systematic investigations have been rare. In *Eriobotrya*, one complete mitogenome of *E. japonica* (MN481990) has been recorded to date [24].

The evolutionary relationship of *E. seguinii* has previously been investigated based on morphology [1,8,18,25,26,27,28,29], inter-simple sequence repeat (ISSR), amplified fragment length polymorphism (AFLP) markers [30,31], internal transcribed spacer (ITS) [12,14,16,32,33,34,35], and genomic data [7,14,17,18,36,37]. However, contradictory evidence from different genomic analyses has confused the relationship between this species and *Eriobotrya*. Initial ITS studies showed that *E. seguinii* (including *E. henryi* and *E. condaoensis*) represents the most basal lineage in *Eriobotrya* [12,32,36]. However, the precise placement of this basal lineage remains contentious and weakly supported: complete nuclear ribosomal DNA (nrDNA) [7] and individual nrDNA loci (18S-5.8S-26S and 26S) [14] have reported *E. seguinii* as sister to the genus *Rhaphiolepis*. Recent studies identified it as sister to core *Eriobotrya* [12,14,16,17,18], whereas organelle (mtDNA and cpDNA) results revealed a strong contradicting signal, either placing this species within *Eriobotrya* [18] or within *Eriobotrya* species, but each species formed different monophyletic clades (*Eriobotrya*-*Rhaphiplepis*) within their respective genera [17] or sister clades within *Rhaphiolepis* [7]. To provide an accurate mitochondrial reference for this pivotal lineage, a well-documented mitogenome from a base lineage species is required. The mitogenomes of this species and the previously reported *E. japonica* (MN481990) were thoroughly compiled and examined. The taxonomic position based on the mitogenome, codon usage, repetitive sequences, and genomic features was carefully investigated in this study. To elucidate the evolutionary history of *E. seguinii*, a phylogenetic analysis was performed using mitogenome data from 19 previously annotated genera of Rosaceae species based on orthologous protein-coding genes. This study characterized the *E. seguinii* mitogenome features, clarified its phylogenetic position, and offered a useful mitogenomic resource for future comparative studies aimed at resolving the broader evolutionary relationships in Maleae.

## 2. Materials and Methods

### 2.1. Plant Materials and Sequencing Data Retrieving

This study utilized a total of 56 mitogenomes (Table S1), representing 19 genera of Rosaceae, including one mitogenome of *E. japonica* acquired from GenBank (accession MN481990) [24]. Raw sequence reads of *E. seguinii* were recently used to determine the phylogenetic relationship between *Eriobotrya* and *Rhaphiolepis* and to extract mitochondrial protein-coding genes [18]. This study attempted to assemble and annotate the complete mitogenome of *E. seguinii*, which represents a distinct method for yielding a novel mitogenomic resource. To obtain genomic data, silica-dried leaves of *E. seguinii* were collected and supplied to Novogene Co., Ltd. (Beijing, China) for DNA extraction. Genomic DNA samples were fragmented using a Covaris LE220R-plus (Covaris, Woburn, MA, USA) to a size of 350 bp. DNA fragments were end-polished, A-tailed, and ligated with a full-length adapter for Illumina sequencing, followed by further PCR amplification. PCR products were purified using the AMPure XP System (Beckman Coulter, Beverly, MA, USA). Subsequently, library quality was assessed on the Agilent 5400 system (AATI) and quantified by real-time PCR (1.5 nM). Qualified libraries were pooled, and paired-end sequencing (PE150) was conducted using the Illumina NovaSeq 6000 sequencing platform (Novogene, Beijing, China), producing 150 bp sequences at both ends and obtained approximately 10 Gb of raw data.

### 2.2. Mitogenome Assembly and Gene Annotation

After sequencing, Fastp version 1.0.1 [38] was used to clean and trim raw reads in paired-end mode using the following parameters: sequence artifacts such as adapter reads (>10 nucleotides aligned to the adapter, permitting ≤ 10% mismatches), unrecognizable bases (exceeding 10% uncertainty in either read), reads shorter than 140 bp (-l 140), and low-quality bases (Phred quality < 5) were eliminated. GetOrganelle v1.7.7 [39] was used to assemble the mitochondrial genome. This program performs iterative seed-based read filtering and de novo graph reconstruction using the *E. japonica* reference mitogenome (MN481990) as the initial seeds. Candidate mitochondrial contigs were identified using three independent features: (i) high and consistent read depth, (ii) a circularized organelle-type assembly graph, and (iii) strong sequence similarity to the reference sequence. We also checked for possible NUMTs and plastid-derived insertions. Contigs showing atypical coverage patterns or lacking mitochondrial similarity were excluded before the final annotation with GeSeq (https://chlorobox.mpimp-golm.mpg.de/geseq.html, accessed on 9 September 2025). The aforementioned data were further reviewed and manually corrected using the CPGview web tool (http://47.96.249.172:16085/cpgview/home; accessed on 15 October 2025) to enhance annotation accuracy. Furthermore, to validate and annotate the open reading frames (ORFs) in *Eriobotrya* mitogenomes, we used ORF Finder (https://www.ncbi.nlm.nih.gov/orffinder/; accessed on 12 January 2026) to identify all possible ORFs ≥ 75 nucleotides, with ATG only as the start codon based on standard mitochondrial genetic code. These ORFs were then translated into their corresponding protein sequences and analyzed at the amino acid level. BLAST searches (https://blast.ncbi.nlm.nih.gov/Blast.cgi; accessed on 12 January 2026) against UniProt (UniProtKB reference proteomes + Swiss-Prot), non-redundant protein database, and tBLASTn, with default parameters, were used to predict the function of ORF proteins. OGDRAW was then used to create a mitogenome map [40].

### 2.3. Repeat Sequence Analysis

The REPuter v1.0 (https://bibiserv.cebitec.uni-bielefeld.de/reputer/, accessed on 16 October 2025) software was used to identify long-term repeat (LTR) sequences, including Reverse (R), Palindromic (P), Forward (F), and Complementary (C) repetitions [41]. The software parameters include a minimum repeat size of 30 bp, a maximum of 5000 bp, and a hamming distance of 3. Simple sequence repeats (SSRs) were identified utilizing the Perl script Microsatellite (MISA) software v2.1 (https://webblast.ipk-gatersleben.de/misa/, accessed on 16 October 2025) [42]. The SSR thresholds were set at 10, 5, 4, 3, 3, and 3, varying from mono- to hexanucleotides. Locates tandem repeats (TRs) in the online tandem repeat finder software (https://tandem.bu.edu/trf/trf.html, accessed on 6 November 2025) using the following standards [43]: Minscore = 50, Maxperiod = 500, Minrepeat = 9, and Match = 2; Mismatch = 7. Identical parameter settings were applied to both *Eriobotrya* species for all repeat detection tools, ensuring a valid quantitative comparison.

### 2.4. Codon Usage

Codon preference analysis was performed on the protein-coding genes of the mitogenome using MEGA (version 11) to compute the relative synonymous codon usage (RSCU) values [44]. The number of codons were counted using DNAsp6 [45]. Codon consumption is more common when the RSCU value exceeds 1.00 and vice versa. The RSCU values for two *Eriobotrya* mitogenomes were grouped according to amino acid properties and then the stacked-bar graph was prepared using R software (version 4.4.2).

### 2.5. Prediction of RNA Editing Events and Nucleotide Diversity

Protein-coding genes were used to predict RNA editing sites in two *Eriobotrya* mitogenomes via the web tool DeepRed-Mt (http://47.96.249.172:16084/deepredmt.html, accessed on 15 October 2025), with a threshold value of 0.9 [46]. Nucleotide diversity values (*Pi*) were calculated using the Perl script method [47]. If the frequencies of the four nucleotides (A, T, G and C) at site *i* were denoted as X_iA_, X_iT_, X_iG_ and X_iC,_ the nucleotide diversity across at *i* site is defined as *Pi* = 1 − (X^2^_iA_ + X^2^_iT_ + X^2^_iG_ + X^2^_iC_). Then, the overall *Pi* was calculated by averaging *Pi* across all *L* sites.

### 2.6. Phylogenetic Analysis

Phylogenetic analysis was performed using the mitogenomes of Rosaceae: one *E. seguinii* from this study, while one *E. japonica* and fifty-five other Rosaceae species retrieved from GenBank, including outgroups *Hemiptelea davidii* (Hance) Planch., and *Hippophae tibetana* Schltdl. The mitochondrial orthologous protein-coding genes shared by all species were extracted using Read2Treee v1.5.3 [48] and aligned by MAFFT v7.0 [49]. For phylogenetic analysis, both Maximum Likelihood (ML) and Bayesian Inference (BI) methods were utilized in Phylosuite v1.2.3 software [50]. The GTR + G + I model was selected after ModelFinder module [51] identified it as the best nucleotide substitution model in IQ-TREE (http://iqtree.cibiv.univie.ac.at/, accessed on 15 October 2025) based on the Bayesian Information Criterion (BIC) score [52]. Phylogenetic trees were reconstructed using Phylosuite software, which integrates ML and BI methods. The nodal support of the ML tree was assessed using SH-aLRT and 1000 ultrafast bootstrap replicates, whereas the BI tree was inferred concurrently for additional topological support. Figtree v1.4.5 (https://github.com/rambaut/figtree/releases/tag/v1.4.5pre, accessed on 21 October 2025) was then used to visualize the tree.

## 3. Results

### 3.1. Genomic Features of the E. seguinii Mitogenome

**Figure 1:**
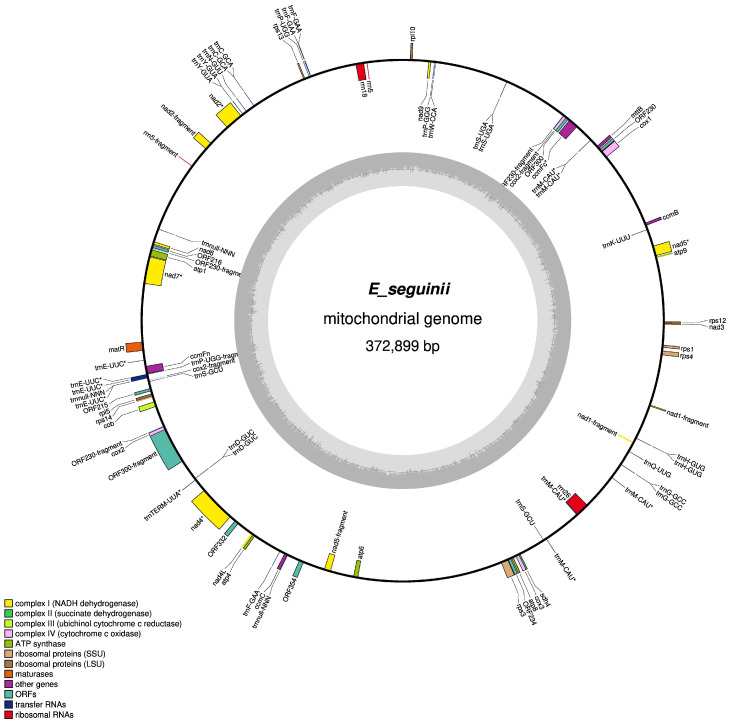
Circular architecture of the *E. seguinii* mitogenome. Gene map showing 71 annotated genes in different functional groups. Features of the transcriptionally clockwise and counter clockwise strands are depicted on the inside and outside of the outer circle, respectively. The inner circle displays the genome coordinates and GC content. Genes from different groups are color-coded for clarity.

The sequenced *E. seguinii* mitogenome had a typical circular architecture, spanning 372,889 bp in length and comprising 71 unique genes, including 47 protein-coding genes (PCGs), 4 ribosomal RNA genes, and 20 transfer RNA genes, with a GC content of 46% (Figure 1). In contrast, the *E. japonica* mitogenome was 434,980 bp long and included 40 PCGs, 3 ribosomal RNA genes, and 15 transfer RNAs, with a GC content of 45.4% (Figure S1 and Table 1). The *E. seguinii* mitogenome consisted of 27.2% A, 22.6% C, 23% G, and 27.2% T.

The *E. seguinii* mitogenome contained 17 core genes, including one maturase gene (*matR*), one ubiquinol cytochrome c reductase gene (*cob*), five cytochrome c oxidase genes (*cox1*, *cox2*, *cox2-fragment* (2), and *cox3*), six ATP synthase genes (*atp8*, *atp4*, *atp6*, *atp1*, *atp9*, and *atp9-fragment*), and four cytochrome C biogenesis genes (*ccmC*, *ccmB*, *ccmFc*, and *ccmFn*). Additionally, the genome has variable genes: 20 transfer RNAs, four ribosomal RNAs (*rrn5*, *rrn18*, *rrn5-fragment* and *rrn26*), one transport membrane protein (*mttB*), one succinate dehydrogenase gene (*sdh4*), 14 NADH dehydrogenase genes (*nad1*-*nad4*, *nad4L*, *nad5*-*nad7*, *nad9*, and a few other fragments), two large ribosomal protein subunits (LSU; *rpl5* and *rpl10*), and six small ribosome protein subunits (SSU; *rps12*, *rrps3*, *rps13*, *ps1*, *rps4*, and *rps14*). Several tRNA genes were encoded by multiple copies, with *trnM-CAU* (*n* = 6), *trnE-UUC* (*n* = 4), *trnF-GAA* and *trnnull-NNN* (*n* = 3). Thirteen genes were found to have intron sequences: nine genes had one intron each; two introns were found in *nad1* and *nad2*, three in *nad4*, and four in *nad7.* Furthermore, 11 unknown ORFs were detected (Table 2), and most hits had low sequence homology (<50% coverage and identity), providing insufficient data to determine their function but calling for further research to investigate these ORFs and their origin. Preliminary analysis showed that in seven out of 11 ORFs, three (*ORF215*, *ORF300* and *ORF332*) were less significantly similar to hypothetical protein, one (*ORF216*) was close to CobW domain-containing (CBWD) protein 1, and three (*ORF230*, *ORF234*, and *ORF354*) showed no significant hits. In addition, four ORF fragments were less than 75 nucleotides and were excluded. Furthermore, tBlastn results showed that five ORFs (*ORF215*, *ORF216*, *ORF300*, *ORF332*, and *ORF354*) were similar to sequences found in *E. japonica* (NCBI) and other Rosaceae genera (such as *Crataegus*, *Pyrus*, *Malus* etc.), while two (*ORF230* and *ORF234)* sequences were not detected in *E. japonica*, but were found in *Crataegus* species (Table S2). In contrast, the *E. japonica* mitogenome contained one duplication (*nad4* (*n* = 2)*)* in the coding gene, but lacked seven coding genes: *cox2-fragment* (747 bp in length), *ORF300-fragment* (855 bp in length), *atp9-frament* (210 bp in length), *nad5-fragment* (102 bp in length), *ndh2-fragment* (547 bp in length), *ORF230-fragment* (243 bp in length), and *ndh1-fragment* (264 bp in length); five tRNAs genes (*trnT-UGU*, *trnTERM-UUA*, *trnnull-NNN (n* = 3), and one rRNA gene (*rrn5-fragment*) were missing, whereas four tRNAs (*trnC-GCA*, *trnF-GAA*, *trnG-GCC* and *trnW-CCA*) were similar in both species, and all others were distinct from each other. Genes contained the following introns: one intron in *ccmFc* and *trns-GCT*; three introns in *nad4*, *nad4-copy2* and *nad5*, and four introns in *nad1*, *nad2* and *nad7* (Table 2).

We further observed divergent mitogenome architectures between the tested species that exhibited gene duplication and fragmentation, such as *nad1* split into 340, 264, and 313 bp and *nad5* split into 1451, 547, and 102 bp in *E. seguinii*, whereas *E. japonica* maintained contiguous gene sequences (*nad1* = 885 and *nad5* = 1995 bp). Furthermore, the *E. seguinii cox2* and *atp9* coding genes contained 1619 bp (one complete 787 bp, one near-complete fragment 747 bp, and one partial fragment 85 bp) and 492 bp (one complete 282 bp, and one near-complete fragment 210 bp), whereas *E. japonica* possessed one complete 787 bp *cox2* and 282 bp *atp9* copies of both genes.

### 3.2. Characteristics of Repeat Sequences

**Figure 2:**
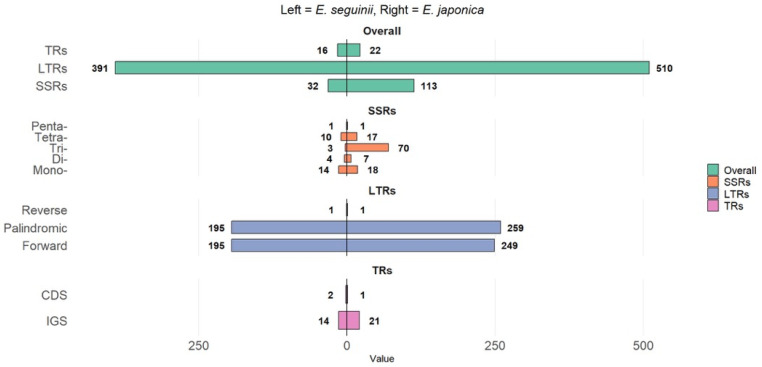
Distribution of repeat elements in the mitogenomes of *E. seguinii* and *E. japonica.* SSRs: simple sequence repeats; LTRs: Long-term repeats; TRs: Tandem repeats; CDS: Coding sequence; IGS: Intergenic space.

SSR loci were identified in the mitogenomes of two *Eriobotrya* species, with an average number of 72.5 SSRs, ranging from 32 (*E. seguinii*) to 113 (*E. japonica*) (Figure 2). The most prevalent SSRs (mono- to pentanucleotides) included mononucleotides in *E. seguinii* (14) and trinucleotides in *E. japonica* (70), followed by tetranucleotides in *E. seguinii* (10), mononucleotides in *E. japonica* (18), and pentanucleotides in both species (1 each). Figure S2 shows that the most common single-base repeat units, A/T repeats, were found in both species (*E. seguinii*: 14; *E. japonica*: 17). Dinucleotide SSRs included AG/CT (*E. seguinii*: 2; *E. japonica*: 5); and trinucleotide SSRs ranged from 1 to 23: AAC/GTT (*E. seguinii:* 1; and *E. japonica* 5), AAG/CTT, AAT/ATT, ACC/GGT, ACG/CGT, ACT/AGT, AGC/CTG, AGG/CCT, ATC/ATG, CCG/CGG (*E. japonica:* 23, 11, 7, 2, 4, 7, 4, 5, 2; respectively), AAAG/CTTT (*E. seguinii*: 2; *E. japonica*: 4), and pentanucleotides (*E. seguinii*: 1; *E. japonica*: 1).

The LTR sequences of the two *Eriobotrya* species varied in length from 391 (*E. seguinii*) to 510 bp (*E. japonica*). Three types of repeats were found; predominantly palindromic and forward repeats, with one reverse repeat identified. *E. japonica* had more forward and palindromic repeats (P: 259; F: 249) than *E. seguinii* (195 for each).

The mitogenomes of *E. seguinii* and *E. japonica* contained 16 and 22 TRs, with 14 to 21 in intergenic regions (IGS) and 1 to 2 in CDS regions, respectively. In Table S3, we observed that the sequence lengths ranged from 8–39 bp in *E. seguinii* to 42 bp in *E. japonica*, with matching degrees from 78 to 100% in *E. seguinii* and 68 to 100% in *E. japonica*. Furthermore, four (25%) to five (22.7%) sequences of *E. seguinii* and *E. japonica* matched perfectly. The copy number of tandem repeats ranged from 1.9 to 4.6, indicating that a considerable fraction of them were incomplete copies. Detectable differences in repeat units, types, and distributions between these two mitogenomes signify the plasticity and structural dynamics of mitochondrial genomes. Thus, they can aid in genomic rearrangement, recombination, and size variations in plant mitogenomes; however, their functional roles remain to be confirmed through further comparative and experimental studies.

### 3.3. Codon Usage Bias Analysis

**Figure 3:**
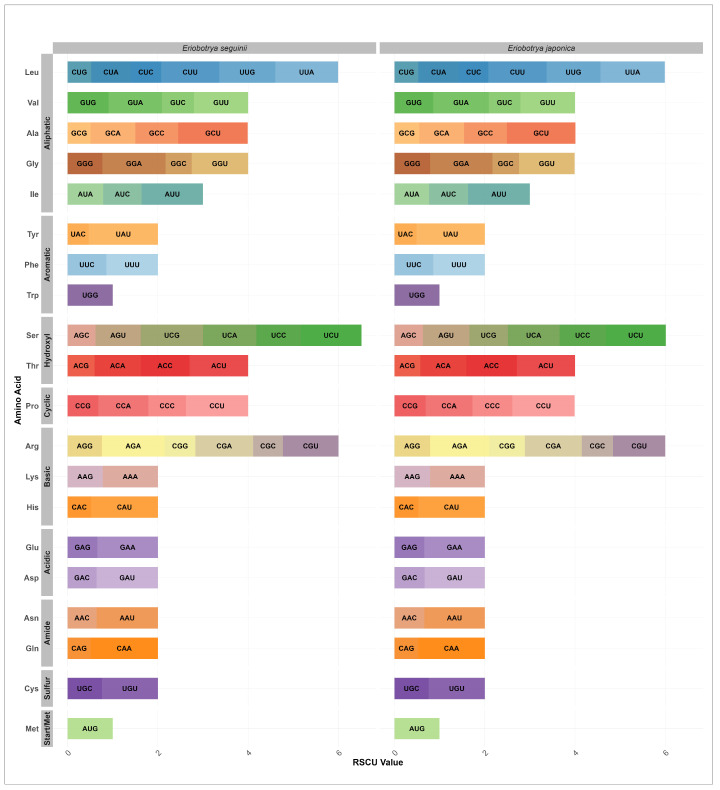
Comparative analysis of relative synonymous codon usage (RSCU) in the mitogenomes of *Eriobotrya* species. Stacked bar plots display the RSCU values for each codon (except STOP codons) organized by amino acid functional groups. Each bar represents the RSCU value for an amino acid partitioned by individual codons. Species comparison showing *E. seguinii* (**left**) and *E. japonica* (**right**). Codon labels within the bars indicate specific nucleotide triplets.

RSCU values were determined using the protein-coding sequences from two *Eriobotrya* mitogenomes. The total number of codons ranged from 10,694 (*E. japonica*) to 13,538 (*E. seguinii*), including termination codons (UAG, UAA, and UGA). Codon usage was nearly consistent across these two mitogenomes, with RSCU values varying between 0 and 2 (Figure 3). Of the 20 amino acids, GCU-encoded alanine was the most prevalent (approximately 1.54), whereas UAC-encoded histidine had the lowest frequency (approximately 0.47). The mitogenomes of the two *Eriobotrya* species showed similar codon preferences representing preserved translational preferences and evolutionary similarity. For instance, alanine (A) had the highest average RSCU values of 1.54 and 1.52, exhibiting a strong preference for GCU. Histidine (Y) also showed a strong preference for UAU codons, with average RSCU values of 1.53 in *E. seguinii* and 1.51 in *E. japonica*, respectively. Glutamate (H) and asparagine (Q) demonstrated strong preferences for codons with maximal RSCU values greater than 1.49 and 1.48, respectively. Additionally, RSCU values were consistent within the group due to the properties of amino acids, which revealed a designed codon bias rather than arbitrary usage. The trend of RCSU values in this study is a typical characteristic observed in plant mitochondrial genomes, which reliably reflects the codon usage pattern in angiosperms. These results indicate that mitochondrial genome features are conserved and exhibit limited species-specific traits in *Eriobotrya*.

### 3.4. RNA Editing Sites and Nucleotide Diversity

**Figure 4:**
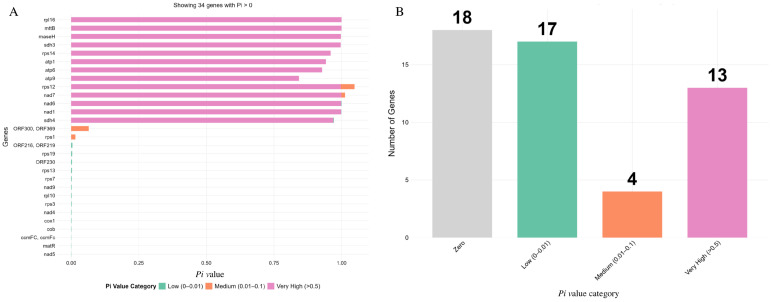
Nucleotide diversity analysis (*Pi*-value) of the complete mitogenomes of *Eriobotrya* species. (**A**) Genes under *Pi* value category from low to high. (**B**) Distribution of gene numbers among the *Pi* value category.

To better understand RNA editing sites and nucleotide diversity (*Pi*), we computed protein-coding sequences from two *Eriobotrya* mitogenomes. Table S4 revealed a total of 658 putative RNA editing sites in *E. seguinii* and 506 in *E. japonica*, which could serve as potential genomic sites for future genetic engineering studies; however, the reported sites are results of in silico prediction and may overestimate true editing events in the absence of experimental validation. The *Pi* values in the coding domain ranged from 0.84 (*atp9*) to 1 (*rpl16* and *mttB*), with an average of 0.246 (Figure 4A,B). Overall, *atp9*, *atp6*, *atp1*, *rps14*, *sdh4*, *sdh3*, *rps12*, *rnaseH*, *nad1*, *nad6*, *nad7*, *rpl16*, and *mttB* had the highest *Pi* values (0.84 to 1.0), which were ten times greater than those of the other core genes (Figure 4A). It can be observed that thirteen genes had Pi values over 0.5 (Figure 4B). These hotspot locations can serve as preliminary candidates for validating DNA barcodes in phylogenetic research to aid in identifying *Eriobotrya* species.

### 3.5. Phylogenetic Analysis

The dataset utilized in this study included 56 species from 19 genera in the Rosaceae family, along with one *Eriobotrya* mitogenome obtained from NCBI and one newly contributed mitogenome. Multiple sequence alignments of the complete mitochondrial genomes resulted in a concatenated dataset of 45,360 aligned matrices containing 614 parsimony-informative (PI) sites, 10,831 invariable sites, and 1102 variable sites, with a GC content of 46.1% and overall nucleotide diversity (*Pi*) of 0.01577. Table 3 provides information on recently identified sequence features.

**Figure 5:**
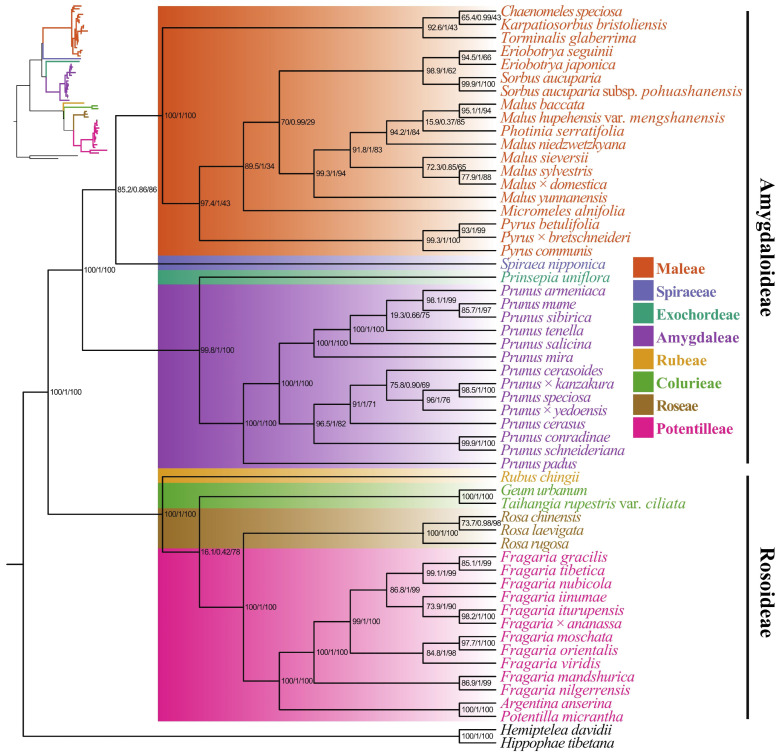
Maximum likelihood (ML) tree of Rosaceae based on mitogenome protein-coding genes. The numbers at the nodes represent ML bootstrap percentages (100 replicates) and Bayesian inference (BI) posterior probabilities (1.0). Rosaceae is divided into eight clades and correspond to the color-legend.

The protein-coding sequences of the mitogenomes elucidated the evolutionary relationships among all genera of Rosaceae, with moderate to high support values (ML: 78–100% and BI-PP: 0.42–1, respectively) and confirmed that *Eriobotrya* species are monophyletic within the Maleae tribe. The basal lineage *E. seguinii* was classified under the genus *Eriobotrya* and has an evolutionary relationship with *E. japonica* (Figure 5).

All Rosaceae genera and the Maleae tribe, each represented by one or more species in the analyses, appear to constitute monophyletic groups (e.g., *Chamaemeles* Lindl., *Eriobotrya* Lindl., *Sorbus* L., *Pyrus* L., *Micromeles* Decne., *Karptiosorbus* Sennikov & Kurtto, *Torminalis* Medik, and *Malus* Mill.). Notably, the Maleae group of Rosaceae, including *Photinia serratifolia*, established a well-supported clade with *Malus* species, with moderate to low support value (ML: 85% and BI-PP: 0.0.37, respectively), and additional sequences from the genus *Photinia* Lindl. will be required to confirm its position within the tribe Maleae.

## 4. Discussion

Despite being the energy source to perform vital cellular functions, plant mitochondrial genomes are more sophisticated than those of animals because of their stable and repeated coding regions [53]. Numerous studies have focused on plastids to clarify the evolutionary relationships across various lineages, given the challenges of acquiring complete sequences of plant mitogenomes; multiple mitogenomes must be examined [54,55]. Most mitogenomes in higher plants exhibit a typical circular architecture, largely due to the presence of extensive repetitive sequences that may facilitate DNA recombination and the formation of subring [56,57]. Usually, mitogenomes of Rosaceae stored in the NCBI database displayed a circular structure with notable size variation, ranging from 270,143 bp (OQ628291) to 535,727 bp (NC_065232); however, a minor variation is seen in GC% [19]. Our constructed mitogenome of *E. japonica* has a circular architecture (Figure S1) and comprises 40 protein-coding genes, 15 tRNAs, and 3 rRNAs (Table 1). However, Yang et al. reported a GC content of 37.80%, with 41 protein-coding genes, 22 tRNAs, and 3 rRNAs [24] based on the same genome data. Additionally, *Eriobotrya* species showed significant variation in genome size, with the *E. seguinii* mitogenome (372,899 bp) being smaller than that of *E. japonica*, differing by 62,080 bp. Despite these size differences, their GC content was generally consistent, ranging from 45.4% in *E. japonica* to 46% in *E. seguinii* in this study compared to the previously reported 37.80% [24]. Notably, the corresponding chloroplast genomes showed consistent lengths for *E. seguinii* (MN577884; 159,450 bp and MN577885; 159,459 bp) with a GC content of 36.7% [7]. These findings provide valuable insights into comparative genome size and mitogenome content.

Plant mitochondrial genomes possess a high degree of gene conservation, numerous non-coding regions, relatively low gene concentration, and instances of RNA editing sites [58]. Mitochondrial DNA encodes tRNAs, rRNAs, and a variable number of ribosomal proteins [59]. This study identified 47 protein-coding genes (PCGs) in the *E. seguinii* mitogenome, exceeding the 40 PCGs reported in *E. japonica* and surpassing most Rosaceae mitogenomes, such as *Prunus salicina* Lindl (*n* = 39). The higher count of PCGs suggests that a small number of ancestral mitochondrial genes might have been transferred from the nuclear genome throughout the evolutionary passages of *E. seguinii*. Forty genes were identified in both *Eriobotrya* mitogenomes; however, a duplicated *nad4* gene was present in *E. japonica* but absent in *E. seguinii*. Rearrangements and horizontal gene transfers within cellular organelles play a vital role in plant evolution resulting in gene acquisition, deletion, and alterations in genome size [60]. This study observed 10 fragments in protein-coding genes (PCGs) of *E. seguinii*, including the *ORF230*, *ORF300*, *atp9*, *cox2*, *nad1*, *nad2*, and *nad5*-fragments, along with one fragment in tRNA (*trnP-UGG*) and one in rRNA (*rrn5*) (Table 2). Markedly, *ORF300-fragment* exhibited the longest mitochondrial protein translation at 855 bp; however, these fragments were absent in *E. japonica*. Similarly, Zhang et al. [21] discovered 22–36 fragments in seven species of *Rosa* L., reporting 14 genes that included 11 PCGs, one rRNA, and two tRNA genes. This phenomenon may explain the unique preservation of functionality in the mitogenome of angiosperms, which acquire tRNA genes through horizontal gene transfer [61]. Interestingly, species within the Rosaceae family possess between two (*Sorbus*) and four (*Geum*, *Photinia*, and *Malus*) ribosomal RNAs in their mitochondrial genomes, confirming the presence of genes essential for environmental adaptability annotated with homologous plastid genes [19,62,63]. In contrast, the *E. seguinii* mitogenome comprises four rRNAs (*rrn5*, *rrn5-fragment*, *rrn18*, and *rrn26*), whereas the *E. japonica* contains three rRNAs (*rrn5*, *rrn18*, and *rrn26*). In the Rosaceae, *Geum*, *Fragaria*, and *Potentilla* lacked *rps12*, whereas five Rosoideae genera lacked *rpl16*, *sdh3*, and *rpl5* [63]. In this study, these genes were also absent in both *Eriobotrya* species, aligning with previous findings. Additionally, these gene deletions may affect the translocation and splicing of mitochondrial genes, potentially altering the development, reproduction, and other physiological and morphological characteristics of plants, such as parasitism, stress responses, stunting, and leaf deformities [64]. In angiosperm mitochondrial genomes, tRNAs are generally not fully represented [65]. In the mitogenomes of *E. seguinii* and *E. japonica*, seven tRNAs (*trnR-TCT*, *trnS-GGA*, *trnP-AGG*, *trnI-GAT*, *trnV-GAC*, *trnT-GGT*, and *trnS-ACT*) were missing, which is a lineage-specific feature of *Eriobotrya*. These genes are general characteristics of the plant mitochondrial genomes. Specifically, *E. seguinii* has three copies of *trnnull-NNN*, four copies of *trnE-UUC*, and six copies of *trnM-CAU*. Both *Eriobotrya* species had three copies of *trnF-GAA*. Furthermore, the *nad4-copy* gene was absent in *E. seguinii* and *cox2-fragment*, *rrn5-fragment* were absent in *E. jaonica*, whereas *trnF-GAA*, *trnW-CCA*, *trnG-GCC*, and *trnC-GCA* were found in both *Eriobotrya* species. Noteworthy, the presence of introns in tRNAs (*trnE-UUC* and *trnM-CAU*) in *E. seguinii* and *trnS-GCT* in *E. japonica* exemplifies the splicing mechanism in mitogenomes as a key stage in tRNA maturation [66]. Furthermore, the mitogenome architecture showed that both species exhibited the rapid, divergent evolution of organelle genomes within a single plant genus.

Frequent SSRs and tandem repeats, in particular, increase genomic diversity, gene duplication, architectural variance, and mitogenome size [67]. SSRs exhibit high polymorphism and cross-species transfer, making them valuable molecular markers in phylogenetic investigations and for analyzing genetic recombination and gene duplication in mitochondrial genomes [68]. These DNA regions, ranging from one to six bp [69], were identified (*E. seguinii*, *32*; *E. japonica*, *113*) in this study at various genomic locations, including intergenic regions (IGS), introns, ORFs, and exons (Figure 2). Our findings indicate that mononucleotide SSRs (*n* = 14) were more common than trinucleotides SSRs (*n* = 10) in the mtDNA of *E. seguinii*, consistent with a previous study in *Prunus* L. [17]. Furthermore, we observed a widespread A/T bias in the repeat units of the *Eriobotrya* mitogenomes (*E. seguinii*: 14; *E. japonica*: 17). The composition of these SSRs, characterized by motifs rich in A and T, substantiates the correlation between SSRs and the AT content of the entire mitogenome [70], and harmonizes with earlier research [71].

“Codon usage bias” denotes notable interspecies variations that influence the selection of specific synonymous codons during protein translation and carry significant evolutionary information in plants [72]. This preference for certain synonymous codons is essential for determining the genetic traits of a species [73]. Our analysis of RSCU values derived from 47 PCGs within the *Eriobotrya* mitogenomes revealed a tendency for codons ending in A/T to be more prevalent, particularly GCU-encoded alanine (RSCU = 1.54, Figure 3). This results are in agreement with previous research on *Rubus* mitogenomes [74], indicating that mitochondrial codon usage preferences are relatively consistent across species.

The nuclear genome sequences (ITS1 and ITS2) of plants exhibit greater variability compared to rRNA genes [75]. In contrast, whole mitogenomes offer unique advantages for studying speciation, population genetics, phylogenetics, and phylogeography. Unlike nuclear or chloroplast genomes, mitogenomes possess distinct characteristics, such as high recombination rates, multi-branch architectures, and variations in gene content, which provide valuable insights into plant evolutionary processes. While nuclear and chloroplast genomes are typically used for species classification and evolutionary analysis, mitogenomes yield additional evolutionary information that is not easily obtained from other organelle genomes. In this study, several genes exhibited *Pi* values ranging from 0.84 to 1.0, as shown in Figure 4. These values were higher than those of other key genes in the mitogenomes, and these genes may serve as suitable molecular markers for future research on evolutionary relationships within the Rosaceae family. Zhang et al. [21] sequenced the *Rosa* mitogenomes to clarify species relationships and developed specific molecular markers for accurate identification based on coding and non-coding regions, highlighting the utility of mtDNA in phylogenetic studies and classification, alongside the important of the nuclear genome. Additionally, we identified 658 potential RNA editing sites in *E. seguinii* and 506 in *E. japonica*, derived from PCGs (Table S2). The significant incidence of RNA editing sites may contribute to differences in sequence variability and gene content among functional PCGs [76].

Many plants exhibit maternal inheritance of their mitochondrial and chloroplast genomes, which facilitates genetic research and makes these organelles the preferred markers for phylogenetic analysis in taxonomy [24]. To understand the genesis, dissemination, feature evolution, and speciation processes within families, a scientifically credible phylogenetic tree must be constructed [77]. Numerous evolutionary incongruences between nuclear, chloroplast, and mitochondrial gene trees have been noted in Rosaceae genera, such as *Eriobotrya* [7,17,18] and *Cotoneaster* Medik. [78], *Potentilla* L. [79], *Taihangia* T.T.Yu & C.L.Li [23], and are often attributed to chloroplast/mitochondrial capture events, ancient hybridization, gene introgression, and incomplete lineage sorting (ILS) [80]. The phylogenetic relationship of *E. seguinii* within *Eriobotrya* has been disputed. Molecular studies have placed it either as sister to the genus *Rhaphiolepis* or within *Eriobotrya*. This study clarifies that *Eriobotrya* species within the Maleae tribe are monophyletic (Figure 5) and confirms that *E. seguinii* belongs to the genus *Eriobotrya*, demonstrating a close evolutionary relationship with *E. japonica*. This result topology aligns with previous research on Rosaceae [81] and is congruent with nuclear data [12,14,16,17,18] while conflicting with chloroplast topologies that group *E. seguinii* with *Rhaphiolepis* [7]. Such discordance is best explained by chloroplast capture via hybridization and introgression. Despite the slow nucleotide substitution rate of plant mitogenomes, their extensive structural variation, large size, frequent genomic rearrangement, and maternal inheritance [82,83] collectively provide lineage-specific phylogenetic signals for resolving nodes, especially in groups with complex histories. Notably, a mitogenome-only phylogeny has inherent limitations; it may exhibit low phylogenetic information and can be influenced by processes such as hybridization and ILS, although it is less common than chloroplast genomes. This study offers a valuable mitogenomic resource for *E. seguinii* by utilizing orthologous protein-coding genes, thereby enhancing our understanding of maternal lineage evolution among Rosaceae species. Further investigation of additional *Eriobotrya* species and mitogenomes is essential for a comprehensive understanding of *Eriobotrya* phylogeny and its evolutionary history in Maleae.

## 5. Conclusions

This study involved the assembly, annotation, and evolutionary lineage of the *E. seguinii* mitogenome, which was compared with previously published *Eriobotrya* mitogenomes. In comparison, even though it exhibited a circular structure, the *E. seguinii* mitogenome was found to be smaller in size than that of *E. japonica*. The mitogenome featured 17 core genes, 11 unknown ORFs genes, and 26 variable genes. It also included 13 genes with intron sequences: nine genes with one intron, two genes with two introns, one gene with three introns, and one gene with four introns. Furthermore, 32 SSRs, including mononucleotides, A/T repeats, AG/CT, AAC/GTT, and AATAG/ATTCT, were the most common. A total of 391 long repetitive sequences, 16 tandem repeats, and 658 potential RNA editing sites were identified in the genome. Ten genes (*sdh4*, *mttB*, *rnaseH*, *nad7*, *nad1*, *nad6*, *rps12*, *atp1*, *ORF230*, and *ORF216/ORF219*) demonstrated the highest Pi values (0.84 to 1.0), which were ten times higher than those of other important genes. Phylogenetic analysis based on mitogenome orthologous protein-coding genes showed that the genus *Eriobotrya* is monophyletic within the tribe Maleae, and *E. seguinii* formed a close evolutionary lineage to *E. japonica* (MN481990). To enrich our understanding of *E. seguinii* genesis and mitochondrial genome-based classification, it is crucial to comprehend the properties of its mitochondrial genome. This study offers a useful mitogenomic resource for future studies on the evolutionary relationships within the Rosaceae family.

## Figures and Tables

**Table 1:** Basic characteristics of complete mitogenomes of *Eriobotrya* taxa.

	*E. seguinii*	*E. japonica*
Total length (bp)	372,899	434,980
Total number of genes (unique)	96 (71)	67 (58; 71 *)
Protein-coding genes	52 (47)	41 (40; 41 *)
rRNA genes	4	3 (3 *)
tRNA genes	40 (20)	23 (15; 22 *)
Genes with intron (s)	13	8
GC content (%)	46	45.4 (37.80 *)
A (%)	27.2	27.3
G (%)	23	22.6
C (%)	22.6	22.9
T (%)	27.2	27.3
GenBank accessions	SRR35934444	MN481990

* indicates result of Yang et al. [24].

**Table 2:** Comparative gene compositions of *Eriobotrya* mitogenomes.

Category	Gene Groups	Genes in *E. seguinii*	Genes in *E. japonica*
Core genes	ATP synthase	*atp1*, *atp4*, *atp6*, *atp8*, *atp9*, ***atp9-fragment*** *	*atp1*, *atp4*, *atp6*, *atp8*, *atp*
	Cytochrome C biogenesis	*ccmB*, *ccmC*, *ccmFc* *, *ccmFn*	*ccmB*, *ccmC*, *ccmFc* *, *ccmFn*
	Ubiquinol cytochrome c reductase	*cob*	*cob*
	Cytochrome c oxidase	*cox1*, *cox2*, ***cox2-fragment (2)***, *cox3*	*cox1*, *cox2*, *cox3*
	Maturase	*matR*	*matR*
Variable genes	ORFs	*ORF215*, *ORF216*, *ORF230*, ***ORF230-fragment (3)***, *ORF234*, *ORF300*, *ORF300-fragment* *, *ORF332*, *ORF354*	*ORF215*, *ORF216*, *ORF230*, *ORF234*, *ORF300*, *ORF332*, *ORF354*
	NADH dehydrogenase	*nad1* **, ***nad1-fragment (2)***, *nad2* **, *nad2-fragment* *, *nad3*, *nad4* ***, *nad4L*, *nad5* *, *nad5-fragment (2)* *, *nad6*, *nad7* ****, *nad9*	*nad1* ****, *nad2* ****, *nad3*, *nad4* ***, ***nad4 (2)*** ***, *nad4L*, *nad5* ***, *nad5-fragment (2)*, *nad6*, *nad7* ****, *nad9*
	Transport membrane protein	*mttB*	*mttB*
	Succinate dehydrogenase	*sdh4*	*sdh4*
	Ribosomal Protein (LSU)	*rpl5*, *rpl10*	*rpl5*, *rpl10*
	Ribosomal Protein (SSU)	*rps1*, *rps3*, *rps4*, *rps12*, *rps13*, *rps14*	*rps1*, *rps3*, *rps4*, *rps12*, *rps13*, *rps14*
	Ribosomal RNAs	*rrn5*, *rrn18*, ***rrn5-fragment***, *rrn26*	*rrn5*, *rrn18*, *rrn26*
	Transfer RNAs	***trnK-UUU***, ***trnS-UGA(2)***, ***trnS-GCU (2)***, *trnW-CCA*, ***trnP-GGG***, ***trnP-UGG***, ***trnP-UGG-fragment***, *trnF-GAA (3)*, *trnC-GCA (2)*, ***trnN-GUU (2)***, ***trnY-GUA (2)***, ***trnE-UUC (4) ^i^***, ***trnD-GUC (2)***, ***trnTERM-UUA***, ***trnnull-NNN(3)***, ***trnM-CAU (6) ^i^***, *trnG-GCC (2)*, ***trnQ-UUG***, ***trnH-GUG (2)***, ***trnT-UGU***	***trnK-TTT***, *trnS-GCT* *, ***trnS-TGA***, *trnW-CCA*, ***trnP-TGG***, *trnF-GAA (3)*, *trnC-GCA*, ***trnN-GTT***, ***trnY-GTA***, ***trnE-TTC (3)***, ***trnD-GTC***, ***trnD-GTC (2)***, ***trnM-CAT (4)***, *trnG-GCC*, ***trnQ-TTG***, ***trnH-GTG***

Notes: Gene *: Gene with one intron; Gene **: Gene with two introns; Gene ***: Gene with three introns; Gene ****: Gene with four introns; Gene *^i^*: one copy has one intron; Numbers in the first bracket indicate the gene copy; Bold text indicates gene present in one genome of *Eriobotrya*.

**Table 3:** Sequence diversity of the *Eriobotrya* mitogenomes.

Sequence Information	Mitochondrial Genome Sequences (mtDNA)
Number of sequences (*n*)	56
Number of *Eriobotrya* species (*n*)	2
Alignment length (bp)	45,360
Invariable sites (bp)	10,831
Variable sites (bp)	1102
Parsimony-information sites (bp)	614
Total number of mutations (bp)	1176
GC contents bp (%)	43.1
Total nucleotide diversity (*Pi*)	0.01577

*n*: Total number, bp: Base Pairs, %: Percent.

## Data Availability

The information about the mitogenome used in this study can be found in the text, and the Rosaceae mitogenomes are provided in the Supplementary Materials (GenBank accession number are provided in Table S1).

## Supplementary Materials

The following supporting information can be downloaded at: https://www.mdpi.com/article/10.3390/genes17010107/s1, Figure S1: The circular architecture of *E. japonica* mitogenome; Figure S2: Comparison of SRR motifs in mitogenomes of *Eriobotrya*. Table S1: Information of mitogenomes of Rosaceae species included in this study. Table S2: Characteristics of ORFs in *Eriobotrya*. Table S3: Tandem repeat distribution across mitogenomes of *Eriobotrya*. Table S4: The number of RNA editing events in two *Eriobotrya* mitogenomes.

## Abbreviations

The following abbreviations are used in this manuscript:

PCGs	Protein-coding genes
tRNA	Transfer RNA
rRNA	Ribosomal RNA
bp	base pair
A	Adenine
T	Thymine
G	Guanine
C	Cytosine
ORFs	Open reading frames
