# Supplementary material for: Assembly, Characterization and Comparative Analysis of the Complete Mitogenome of Small-Leaved Eriobotrya seguinii (Maleae, Rosaceae)
Source: Genes (Basel). 2026 Jan 20;17(1):107. doi: 10.3390/genes17010107 (PMC12841229; doi:10.3390/genes17010107)
Supplement: Supplementary file 1 [file genes-17-00107-s001.zip › Table S3.pdf]

Table S3. Tandem repeat distribution across mitogenomes of *Eriobotrya*

Species	Indices	Location	Period Size	Copy Number	Consensus Size	Percent Matches	Percent Indels	Score	A	C	G	T	Entropy (0-2)
E. japonica	494--546	IGS	24	2.2	24	96	0	97	7	41	20	30	1.8
	36002--36044	IGS	18	2.6	16	82	10	52	25	32	20	20	1.97
	44543--44621	IGS	39	2	39	95	0	140	21	29	18	30	1.97
	80922--80947	IGS	13	2	13	100	0	52	53	0	23	23	1.46
	99505--99556	IGS	21	2.5	21	100	0	104	28	38	5	26	1.79
	101270--101305	IGS	15	2.4	15	100	0	72	47	0	30	22	1.52
	132596--132635	IGS	8	4.6	8	82	17	53	50	0	2	47	1.14
	132588--132630	IGS	19	2.3	19	100	0	86	53	0	4	41	1.21
	138296--138348	IGS	24	2.2	24	96	0	97	7	41	20	30	1.8
	160242--160283	IGS	21	2	20	86	4	57	14	42	4	38	1.66
	165405--165503	IGS	30	3.1	30	71	20	101	19	15	9	56	1.65
	165423--165533	IGS	42	2.6	42	68	13	102	18	14	9	56	1.65
	165435--165490	IGS	18	3.1	18	77	10	60	17	16	8	57	1.64
	165453--165513	IGS	24	2.5	24	83	0	68	21	13	6	59	1.57
	165515--165551	IGS	18	2.1	18	94	0	65	5	21	16	56	1.59
	262144--262239	IGS	30	3.2	30	64	21	83	26	13	25	35	1.93
	284272--284314	CDS (rrn26)	18	2.4	18	81	18	54	34	25	20	18	1.96
	293793--293829	IGS	17	2.2	17	90	0	56	43	18	8	29	1.79
	340444--340481	IGS	20	1.9	20	89	10	60	23	10	34	31	1.89
	351392--351426	IGS	18	1.9	18	100	0	70	17	31	11	40	1.85
	375236--375289	IGS	22	2.5	23	85	8	69	14	48	9	27	1.75
	404262--404295	IGS	14	2.5	14	90	4	52	23	14	38	23	1.92
E. seguinii	20954--20979	IGS	13	2	13	100	0	52	53	0	23	23	1.46
	39548--39599	IGS	21	2.5	21	100	0	104	28	38	5	26	1.79
	45633--45672	IGS	8	4.6	8	82	17	53	47	2	0	50	1.14
	45638--45680	IGS	19	2.3	19	100	0	86	41	4	0	53	1.21

74596--74649	IGS	22	2.5	22	85	8	67	27	9	48	14	1.75
74613--74657	IGS	21	2.1	22	87	4	65	26	15	37	20	1.92
98454--98493	IGS	18	2.5	15	78	21	53	40	12	32	15	1.84
98459--98493	IGS	18	1.9	18	100	0	70	40	11	31	17	1.85
109404--109441	IGS	20	1.9	20	89	10	60	31	34	10	23	1.89
216049--216090	ORF300-fragment-intron	21	2	20	86	4	57	38	4	42	14	1.66
237984--238036	IGS	24	2.2	24	96	0	97	30	20	41	7	1.8
246066--246108	IGS	18	2.6	16	82	10	52	20	20	32	25	1.97
324441--324483	CDS (rrn26)	18	2.4	18	81	18	54	34	25	20	18	1.96
333962--333998	IGS	17	2.2	17	90	0	56	43	18	8	29	1.79
352900--352978	IGS	39	2	39	95	0	140	30	18	29	21	1.97
370146--370179	IGS	14	2.5	14	90	4	52	23	14	38	23	1.92
