# Supplementary figures and images for: Assembly, Characterization and Comparative Analysis of the Complete Mitogenome of Small-Leaved Eriobotrya seguinii (Maleae, Rosaceae)
Source: Genes (Basel). 2026 Jan 20;17(1):107. doi: 10.3390/genes17010107 (PMC12841229; doi:10.3390/genes17010107)

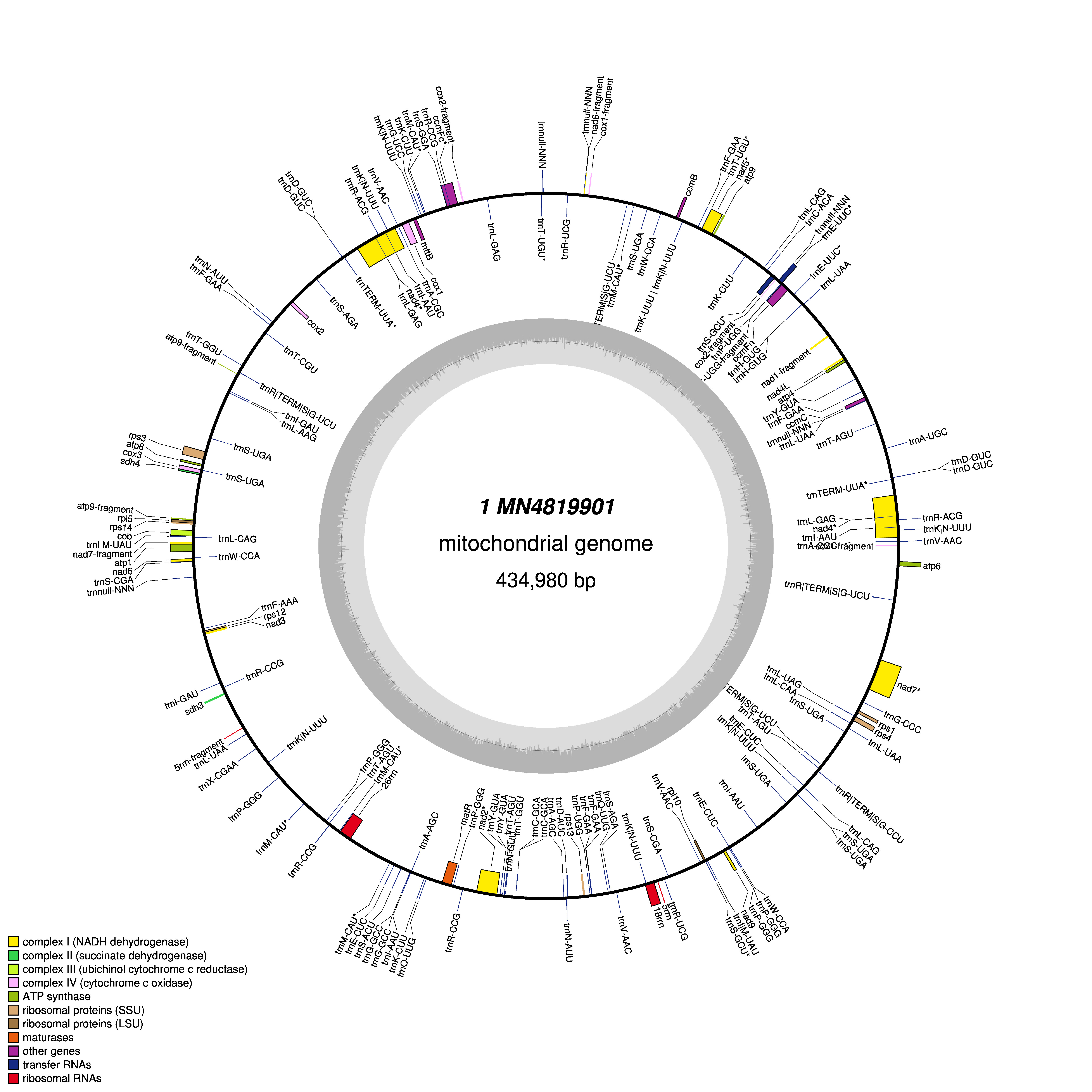

Supplement: Supplementary file 1 [file genes-17-00107-s001.zip › Figure S1.jpg]

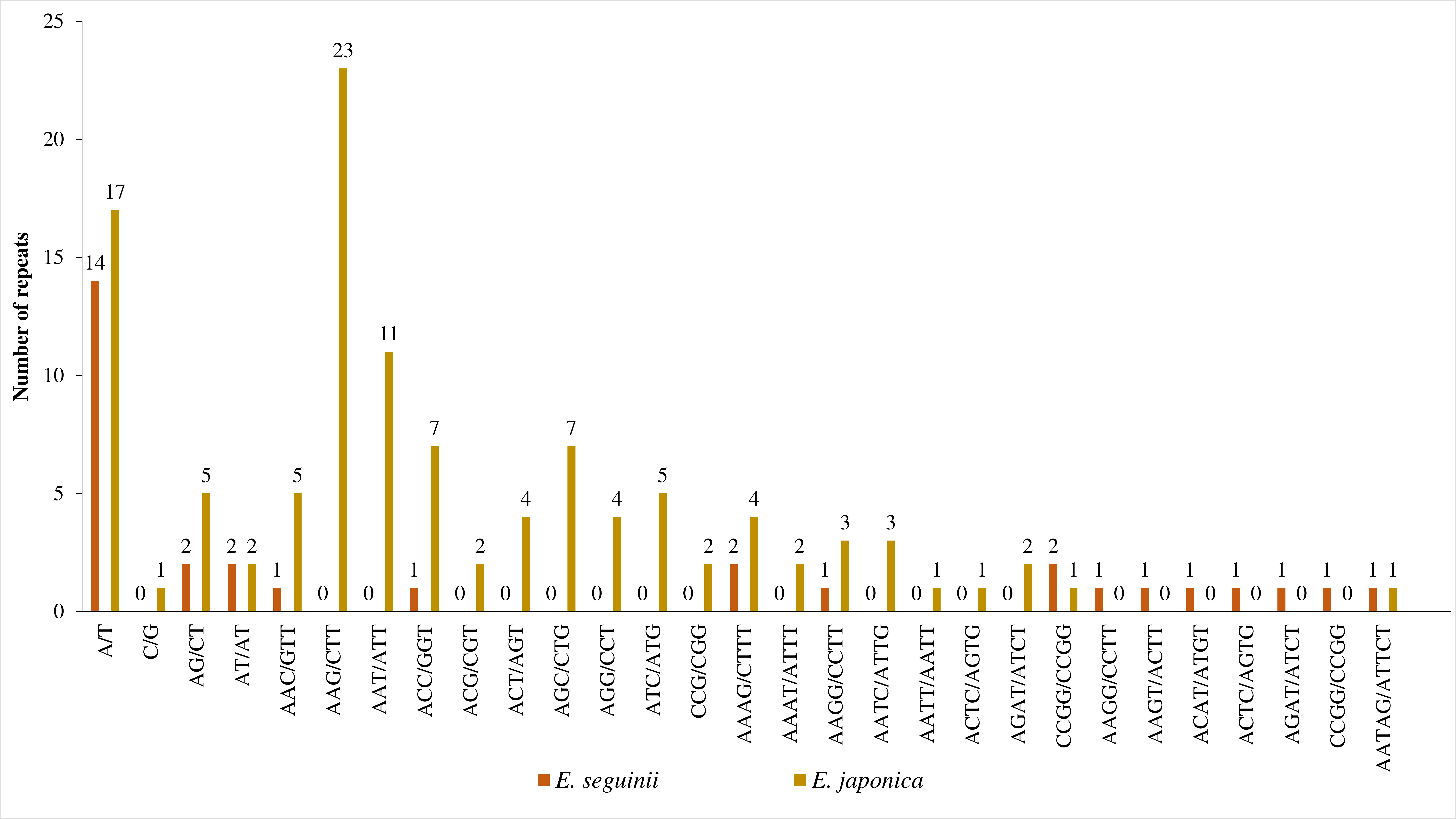

Supplement: Supplementary file 1 [file genes-17-00107-s001.zip › Figure S2.jpg]
